# Plant Disease Control by Non-Thermal Atmospheric-Pressure Plasma

**DOI:** 10.3389/fpls.2020.00077

**Published:** 2020-02-14

**Authors:** Bhawana Adhikari, Kamonporn Pangomm, Mayura Veerana, Sarmistha Mitra, Gyungsoon Park

**Affiliations:** ^1^Plasma Bioscience Research Center, Kwangwoon University, Seoul, South Korea; ^2^Department of Basic Science, Maejo University Phrae Campus, Phrae, Thailand

**Keywords:** atmospheric-pressure plasma, plasma-treated water, decontamination, disinfection, plant pathogens

## Abstract

Disease stresses caused by pathogenic microorganisms are increasing, probably because of global warming. Conventional technologies for plant disease control have often revealed their limitations in efficiency, environmental safety, and economic costs. There is high demand for improvements in efficiency and safety. Non-thermal atmospheric-pressure plasma has demonstrated its potential as an alternative tool for efficient and environmentally safe control of plant pathogenic microorganisms in many studies, which are overviewed in this review. Efficient inactivation of phytopathogenic bacterial and fungal cells by various plasma sources under laboratory conditions has been frequently reported. In addition, plasma-treated water shows antimicrobial activity. Plasma and plasma-treated water exhibit a broad spectrum of efficiency in the decontamination and disinfection of plants, fruits, and seeds, indicating that the outcomes of plasma treatment can be significantly influenced by the microenvironments between plasma and plant tissues, such as the surface structures and properties, antioxidant systems, and surface chemistry of plants. More intense studies are required on the efficiency of decontamination and disinfection and underlying mechanisms. Recently, the induction of plant tolerance or resistance to pathogens by plasma (so-called “plasma vaccination”) is emerging as a new area of study, with active research ongoing in this field.

## Introduction

The control of plant diseases is essential for sustainable food crop production. Many plant disease management strategies have played significant roles in reducing the frequency and intensity of diseases. However, 20%–30% loss of crop production still occurs annually because of plant diseases (Oerke, 2006). Particularly, the current global climatic change makes disease control more complicated and difficult. The elevation of temperature and CO_2_ concentration can change the susceptibility of plants to specific pathogens, leading to the emergence of new diseases (Elad and Pertot, 2014).

Under the current circumstances, traditional disease management strategies have often shown limitations in control efficiency and have multiple drawbacks. Chemical-based methods are most frequently applied for plant disease control; however, the emergence of pathogen resistance and environmental pollution make this strategy less reliable. The induction of plant resistance through species breeding and genetic engineering has been considered as a stable and long-lasting method for plant disease control. Public view on genetically modified organisms is still a barrier for popular use. Biocontrol is the most environmentally friendly method, but the control efficiency in the field needs to be improved.

Disease management in modern agriculture should include considerations from various points of view. Integrative approaches and technologies are one of those considerations for efficient plant disease control strategies. As a potential alternative, atmospheric-pressure non-thermal plasma has recently received attention in managing plant diseases. The antimicrobial activity of atmospheric-pressure non-thermal plasma has been most frequently demonstrated in many studies (for review, Pignata et al., 2017; Sakudo et al., 2019). Owing to its efficient antimicrobial activity, plasma can be usefully applied to eradicate microbes that are pathogenic to plants. Reports on the control of plant diseases by plasma are currently being accumulated (Ito et al., 2017). The majority of studies demonstrate either the efficient inactivation of plant pathogenic microbes *in vitro* or the disinfection of seeds and plants. In this review, we overviewed the case studies of plant disease control by plasma against plant pathogenic microbes cultured *in vitro* and those associated with seeds or plants. In addition, the mechanisms of plasma action are discussed. Owing to the limitation of length, literature particularly related to plant pathogenic microbes was included in this review.

## Atmospheric-Pressure Non-Thermal Plasma Technology

Plasma is an ionized gas, often referred to as the fourth state of matter (Compton and Langmuir, 1930). When high energy is applied to gas, it transforms into the plasma state. In reality, the plasma state can be produced by applying a high electric voltage to gas. Reactive species, charged species, UV radiation, free electrons, and an electric field are generated in the plasma state, and these components can greatly affect the biological processes. Since non-thermal plasma can be produced at atmospheric pressure, its biological application has been actively studied in the fields of medicine and agriculture (for review, Adamovich et al., 2017; Ito et al., 2017). Plasma has frequently been demonstrated to be effective in microbial inactivation and cancer cell death. In contrast, plasma can also enhance cell differentiation, wound healing, seed germination, and plant growth. These two contrasting effects (inactivation and activation) are often observed as a consequence of the plasma dose applied.

## Inactivation of Phytopathogenic Microorganisms in Culture Suspension by Plasma

Numerous studies have demonstrated the antimicrobial activity of non-thermal atmospheric-pressure plasma on plant pathogenic microorganisms, mostly focusing on bacteria and fungi (for review, Ito et al., 2017). In those studies, plasma treatment was performed on microbial culture suspension or contaminated food sources, plant bodies, and seeds. In this section, we first discuss the effect of the plasma treatment on the phytopathogenic microbial cell suspension (**Tables 1** and **2**).

**Table 1:** Control of bacterial pathogens by atmospheric-pressure non-thermal plasma.

Treated object	Target bacteria	Plasma types	Reference
Suspension culture	*Erwinia carotovora*	Gliding arc discharge	Moreau et al., 2007
Post-harvest grape and banana	Bacteria	High-field plasma	Liu et al., 2011
Post-harvest almonds	*Salmonella anatum* *Salmonella enteria* serovar Stanley *Salmonella enteritidis* *Escherichia coli*	Atmospheric-pressure plasma jet	Niemira, 2012
Post-harvest corn salad leaves	*E. coli* K12	Atmospheric-pressure plasma jet	Baier et al., 2013
Post-harvest lettuce, tomato, carrot	*E. coli*	Argon atmospheric-pressure cold plasma	Bermudez-Aguirre et al., 2013
Tomato seeds	*Ralstonia solanacearum*	Helium plasma	Jiang et al., 2014
Post-harvest strawberry (in package)	Mesophilic bacteria	Atmospheric cold plasma	Misra et al., 2014
Suspension culture	*Erwinia amylovora*	Gliding arc discharge plasma	Mráz et al., 2014
Post-harvest cherry, tomato, and strawberry	*E. coli* *Salmonella* *Listeria monocytogenes*	Dielectric barrier discharge (DBD) atmospheric cold plasma	Ziuzina et al., 2014
Post-harvest whole black peppers	*Bacillus subtilis* *Bacillus atrophaeus* *Salmonella enterica*	Radio frequency plasma jet Microwave-generated plasma	Hertwig et al., 2015
Post-harvest cabbage, lettuce, and figs	*Salmonella typhimurium* *L. monocytogenes*	Microwave-powered cold plasma	Lee et al., 2015
Onion, radish, cress, and alfalfa seeds	*E. coli*	Atmospheric-pressure volumetric DBD plasma	Butscher et al., 2016a
Wheat seeds	*Geobacillus stearothermophilus*	Atmospheric-pressure DBD plasma	Butscher et al., 2016b
Post-harvest romaine lettuce	*E. coli* O157:H7 *Salmonella* *L. monocytogenes*	DBD atmospheric cold plasma	Min et al., 2016
Cruciferous seeds	*Xanthomonas campestris*	Low-pressure plasma	Nishioka et al., 2016
Post-harvest radicchio leaf	*E. coli* O157:H7 *L. monocytogenes*	DBD atmospheric cold plasma	Pasquali et al., 2016
Hydroponic solution	*R. solanacearum*	Discharge plasma reactor	Okumura et al., 2016
Post-harvested grape tomato, spinach, and cantaloupe	*E. coli* O157:H7 *S. typhimurium* *Listeria innocua*	Cold plasma-activated hydrogen peroxide aerosol	Jiang et al., 2017
Broccoli seeds	*Bacillus cereus* *E. coli* *Salmonella* spp. *Staphylococcus aureus* *L. monocytogenes*	Corona discharge plasma jet	Kim et al., 2017
Rice seeds	*Burkholderia plantarii*	Atmospheric-pressure plasma jet	Ochi et al., 2017
Suspension culture	*X. campestris* pv. *campestris*	Roller conveyer atmospheric-pressure plasma	Toyokawa et al., 2017
Post-harvest almonds	*E. coli* *Salmonella* spp. *Shigella*	Gliding arc non-thermal plasma	Fatemeh et al., 2018
Post-harvest perilla leaves	*E. coli* *S. aureus*	Cylinder-type DBD plasma with underwater bubbler	Ji et al., 2018
Post-harvest black peppers	*B. subtilis* *E. coli* *S. enteritidis*	Diffuse coplanar surface barrier discharge plasma	Mošovská et al., 2018
Suspension culture	*Clavibacter michiganensis* subsp. *sepedonicus* *Dickeya solani* *X. campestris* pv. *campestris* *Pectobacterium atrosepticum* *Pectobacterium carotovorum* subsp. *carotovorum*	Glow discharge plasma	Motyka et al., 2018
Post-harvest kumquat fruits	*S. aureus* *Salmonella* spp. *B. cereus*	Intermittent corona discharge plasma jet	Puligundla et al., 2018b
Post-harvest lettuce and sprout	*E. coli* K12 *Pseudomonas fluorescens* *Pseudomonas marginalis* *P. carotovorum* *L. innocua*	Microwave plasma processed air	Schnabel et al., 2018
Lentil seeds	*E. coli* *L. monocytogenes* *S. enterica* *S. aureus* *G. stearothermophilus*	Diffuse coplanar surface barrier discharge plasma	Waskow et al., 2018
Post-harvest mung bean sprout	Aerobic bacteria	Plasma-activated water	Xiang et al., 2019

**Table 2:** Control of fungal pathogens by atmospheric-pressure non-thermal plasma.

Treated object	Target fungi	Plasma types	Reference
Fungal spore suspension	*Aspergillus niger* *Penicillium citrinum*	Microwave-induced argon plasma	Park et al., 2003
Fungal spore suspension	*A. niger* *Cladosporium cladosporioides* *P. citrinum* *Chaetomium* sp.	Microwave-induced argon plasma	Park et al., 2004
Fungal spore suspension	*A. niger* *P. citrinum*	Hydrogen releasing atmospheric-pressure plasma	Nojima et al., 2007
Post-harvest nuts	*Aspergillus parasiticus*	Low-pressure cold plasma	Basaran et al., 2008
Grains and legume seeds	*Aspergillus* spp. *Penicillium* spp.	Low-pressure plasma	Selcuk et al., 2008
Fungal spore suspension	*Ascochyta pinodella* *Fusarium culmorum*	Atmospheric-pressure dielectric barrier discharge (DBD) plasma	Avramidis et al., 2010
Fungal spore suspension	*Penicillium digitatum*	High-density non-equilibrium atmospheric-pressure plasma	Iseki et al., 2011
Post-harvest grape and banana	Fungi	High-field plasma	Liu et al., 2011
Fungal spore suspension	*Fusarium graminearum* *Fusarium oxysporum*	Microwave plasma jet	Na et al., 2013
Post-harvest rice and lemon	*Aspergillus oryzae* *P. digitatum*	Atmospheric-pressure plasma	Hayashi et al., 2014
Rice seeds	*Fusarium fujikuroi*	Atmospheric-pressure non-thermal DBD plasma	Jo et al., 2014
Fungal spores and tomato seeds	*Cladosporium fulvum*	Atmospheric-pressure plasma jet	Lu et al., 2014
*Cicer arietinum* seeds	Infected microbes	Surface micro-discharge plasma FlatPlaSter 2.0	Mitra et al., 2014
Brassicaceous seeds	*Rhizoctonia solani*	Atmospheric- and low-pressure plasma	Nishioka et al., 2014
Plant leaves	*Colletotrichum gloeosporioides*	Atmospheric-pressure plasma jet	Zhang et al., 2014
Fungal spore suspension	*F. oxysporum*	Micro DBD plasma	Panngom et al., 2014
Barley and corn seeds	Infected fungi	Glow discharge plasma	Braşoveanu et al., 2015
Fungal spore suspension	*P. digitatum*	Flux-defined atmospheric-pressure oxygen radical source	Hashizume et al., 2015
Rice seeds	*F. fujikuroi*	Arc discharge plasma	Kang et al., 2015
Wheat seeds	*Alternaria alternata* *Alternaria botrytis* *Alternaria brasiliensis* *F. culmorum* *Furasium oxysporum* *Gibberella avenacea* *Gibberella intricans* *Gibberella zaea* *Penicillium* spp. *Rhizopus stolonifera* *Trichoderma* spp. Non–spore-forming fungi	Low-temperature plasma	Kordas et al., 2015
Post-harvest blueberries	Contaminated fungi	Atmospheric-pressure cold plasma	Lacombe et al., 2015
Post-harvest date palm fruit	*A. niger*	Double atmospheric-pressure cold plasma	Ouf et al., 2015
Post-harvest maize	*Aspergillus* spp.	Atmospheric-pressure fluidized bed plasma	Dasan et al., 2016a
Post-harvest hazelnuts	*Aspergillus flavus* *A. parasiticus*	Atmospheric-pressure fluidized bed plasma	Dasan et al., 2016b
Rice seeds	Infected fungi	Atmospheric hybrid micro corona discharge plasma	Khamsen et al., 2016
Post-harvest pistachio nuts	*A. flavus*	Cold plasma streamer	Sohbatzadeh et al., 2016
Post-harvest citrus	*P. digitatum*	Atmospheric-pressure DBD plasma	Yagyu et al., 2016
Wheat seeds	*Fusarium nivale* *F. culmorum* *Trichothecium roseum* *A. flavus* *Aspergillus clavatus*	Diffuse coplanar surface barrier discharge plasma	Zahoranova et al., 2016
Basil seeds	Infected fungi	Surface DBD plasma	Ambrico et al., 2017
Post-harvest hazelnuts	*Aspergillus* spp.	Atmospheric-pressure fluidized bed plasma	Dasan et al., 2017
Broccoli seeds	Infected molds and yeasts	Corona discharge plasma jet	Kim et al., 2017
Rice seeds	*F. fujikuroi*	Atmospheric-pressure plasma	Ochi et al., 2017
Cucumber and pepper seeds	*Didymella bryoniae* *Didymella licopersici* *Cladosporium cucumerinum*	Diffuse coplanar surface barrier discharge plasma	Stepanova et al., 2017
Post-harvest mandarin fruit	*Penicillium italicum*	Atmospheric-pressure cold plasma	Won et al., 2017
Barley and wheat seeds	*Penicillium verrucosum*	High-voltage DBD plasma	Los et al., 2018
Soybean seeds	*Diaporthe/Phomopsis*	Atmospheric-pressure DBD plasma	Pérez Pizá et al., 2018
Pak choi seeds	Infected fungi	Corona discharge plasma jet	Puligundla et al., 2018a
Post-harvest kumquat fruits	*P. digitatum*	Intermittent corona discharge plasma jet	Puligundla et al., 2018b
Lentil seeds	*Penicillium decumbens* *A. niger*	Diffuse coplanar surface barrier discharge plasma	Waskow et al., 2018
Pine seeds	*Fusarium circinatum*	Diffuse coplanar surface barrier discharge plasma	Sera et al., 2019
Fungal spore suspension	*C. gloeosporioides*	Atmospheric-pressure corona plasma-activated water	Wu et al., 2019
Post-harvest mung bean sprout	Infected fungi	Plasma-activated water	Xiang et al., 2019
Maize seeds	*A. flavus* *A. alternata* *F. culmorum*	Diffuse coplanar surface barrier discharge plasma	Zahoranova et al., 2019

### Bacterial Pathogens

Although causing a smaller number of diseases and relatively less economic damage than fungi or viruses, plant pathogenic bacteria have a great negative impact on the economic condition in many agricultural countries. Approximately 150 species of bacteria have been found to cause plant diseases. Bacterial pathogens are transferred by biological vectors, such as insects and weeds, and physical factors, such as wind and rain, under field conditions (Agrios, 1997). These pathogens invade plants either through natural openings, such as the stomata and lenticels, or by wounding and moving inside the plant through the xylem (Akhavan et al., 2013). Disease symptoms appear in the form of specks, spots, blights, vascular wilts, tumors, rots, and cankers on the leaves, flowers, fruits, stems, roots, and tubers of affected plants (Horst, 2001); a set of pathogenicity genes are responsible for the establishment of a disease (Salmond, 1994; van Baarlen et al., 2007).

Atmospheric-pressure non-thermal plasma has demonstrated antibacterial potential by inhibiting and reducing the growth of bacteria (Mai-Prochnow et al., 2014; Gilmore et al., 2018). The majority of studies have been focused on food-contaminating and -poisoning bacteria, such as *Escherichia coli*, *Staphylococcus aureus*, *Listeria*, and *Salmonella* (Baier et al., 2013; Sun et al., 2014; Baier et al., 2015; Matan et al., 2015). Compared to that, the treatment of bacteria causing plant diseases has been relatively less explored in the application of plasma (**Table 1**). Several studies demonstrate that plasma treatment of phytopathogenic bacterial suspension can reduce the number of viable bacteria in a time-dependent manner (Moreau et al., 2007; Mráz et al., 2014; Toyokawa et al., 2017; Motyka et al., 2018). Gliding arc discharge can effectively inactivate the potato pathogen *Erwinia carotovora* by altering the bacterial membrane (Moreau et al., 2007). Plasma exposure on two plant pathogenic bacteria, *Clavibacter michiganensis* (Gram-positive) and *Erwinia amylovora* (Gram-negative), decelerated their growth and reproduction (Mráz et al., 2014). Recently, the direct contact of bacterial suspensions with direct current (DC)-based glow discharge plasma was found to rapidly eradicate phytopathogenic bacteria, such as *Clavibacter michiganensis* subsp. *sepedonicus*, *Dickeya solani*, *Xanthomonas campestris* pv. *campestris*, *Pectobacterium atrosepticum*, and *Pectobacterium carotovorum* subsp. *carotovorum* (Motyka et al., 2018). The viability of *X. campestris* pv. *campestris* placed on a roller plasma conveyer was decreased, showing significant degradation of lipopolysaccharides and oxidation of genomic DNA (Toyokawa et al., 2017). The pathogenicity and colony forming unit of *Ralstonia solanacearum* were significantly reduced after the discharge plasma treatment of a hydroponic solution (Okumura et al., 2016). Plasma processed air (PPA) is a novel concept that can be used in the food packaging industry to reduce the load of microbes on packaging material and fresh produce. PPA treatment for 5 min reduced the contamination of PET packaging material with *Pseudomonas fluorescens* and *P. carotovorum* by 2 log_10_ cfu ml^−1^. A reduction higher than 5 log_10_ cfu ml^−1^ was observed for *P. carotovorum* contamination on sprouts (Schnabel et al., 2018). These studies show no specificity between food and plant pathogenic bacteria with respect to the bactericidal effect of plasma. In addition, plasma treatment can efficiently reduce the plant pathogenic bacterial contamination in water or waste, which may act as a source of plant disease inoculum. However, plasma often induces the sub-lethal state of bacteria to the viable but non-culturable (VBNC) state (Xu et al., 2018); thus, intense research is still needed for complete eradication.

Non-thermal plasma produces many reactive oxygen and nitrogen species (RONS), which are toxic to bacterial pathogens at high concentrations. These RONS oxidize proteins, lipids, and nucleic acids and lead to pathogen destruction. They also drive epigenetic regulation, which might abolish bacterial pathogenicity (Casadesus and Low, 2006). A detailed mechanism of plasma action is discussed later in this review.

### Fungal Pathogens

Non-thermal plasma has been frequently applied to inactivate fungal pathogens during food decontamination (for review, Misra et al., 2019). In these studies, the potentiality of plasma is well described in inactivating fungal spores and eradicating mycotoxins. In contrast, the inactivation of plant pathogenic fungi by plasma has been relatively less reported than that of food-spoiling fungi. The plasma-mediated eradication of fungal spores can prevent disease development after germination, inoculum spread, and post-harvest diseases. The efficient inactivation of spores in suspension by plasma has been demonstrated in several phytopathogenic fungi such as *Aspergillus, Penicillium, Fusarium, Cladosporium, Phomopsis, Colletotrichum, Ascochyta, Chaetomium*, and *Rhizoctonia* (see **Table 2**). In these studies, various plasma sources using different combinations of gases were used to eradicate fungal spores submerged in liquid culture media or water under laboratory conditions.

Many studies showed efficient fungal spore eradication by dielectric barrier discharge (DBD)-type plasma (Nojima et al., 2007; Avramidis et al., 2010; Iseki et al., 2011; Panngom et al., 2014; Hashizume et al., 2015). Particularly, Nojima et al. (2007) developed a plasma device releasing atomic hydrogen and showed the deactivation of fungal pathogens and the neutralization of harmful OH radicals in the air by the atomic hydrogen released from plasma. In this study, O and OH radicals generated by plasma were not major players in inactivating fungal spores in the air because of their short lifetime. Atomic hydrogen produced from the plasma device was suggested to be critical for inactivating fungal spores. A hydrogen atom surrounded by water molecules, H^+^(H_2_O)_m_, has a long lifetime (3–5 s in air) and reacts with O_2_ and O_2_^−^(H_2_O)_n_ in the air, forming HO_2_ or HO_2_^−^, which are very reactive and thus can cause damage to fungal spores. However, the authors also found an additional effect of atomic hydrogen; it alleviates the toxicity of OH radicals in the air to the user. This study was a good example that key plasma factor(s) responsible for antimicrobial activity can vary depending on the condition and plasma sources. The plasma-treated fungal spores often show severe morphological degeneration and seem to undergo necrotic death. However, one study demonstrated the possibility that the fungal spores treated with micro DBD plasma can undergo apoptosis-like death without severe morphological destruction (Panngom et al., 2014). Besides fungal spores, fungal hyphae were occasionally the target of plasma treatment (Avramidis et al., 2010). Plasma can also be produced using microwave power without electrodes, and the microwave-induced plasma can destroy fungal spore cells and inhibit subsequent hyphal growth of *Penicillium citricum*, *Fusarium graminearum*, and *F. oxysporum* (Park et al., 2003; Park et al., 2004; Na et al., 2013).

Plasma-treated water also exhibits anti-fungal activity. Wu et al. (2019) demonstrated that the air and oxygen plasma-activated water (PAW) deactivated the spores of *C. gloeosporioides*. In their study, the air PAW was more efficient than the oxygen PAW, and the long-lived species, such as nitrate and ozone, generated in PAW might play a critical role in this anti-fungal effect (Wu et al., 2019).

### Viral Pathogens

Compared to bacterial and fungal phytopathogens, plant viruses have hardly been examined in terms of plasma application. The antiviral effects of plasma have been occasionally demonstrated in human and animal viruses, as well as bacteriophages (Zimmermann et al., 2011; Aboubakr et al., 2015; Guo et al., 2018). The inactivation of plant viruses by plasma was demonstrated in a recent study (Hanbal et al., 2018). In this study, inoculation with plasma irradiated tobacco mosaic virus (TMV) solution did not induce the development of disease in tobacco plants, whereas necrotic local lesions developed on tobacco leaves inoculated with non-irradiated TMV solution (Hanbal et al., 2018). Min et al. (2016) also showed that viral load was significantly reduced when romaine lettuce inoculated with Tulane virus was treated with DBD plasma.

## Plasma Application in Disease Control

### Seed-Borne Diseases

Seed contamination with pathogenic microorganisms can cause diseases in seedlings, leading to a significant reduction in crop yield. In addition, the pathogens preserved in seeds can be a source of disease propagation. Since many seed-borne diseases are associated with the infection of a flowering part during the pre-harvest period, the pre-harvest control of diseases might be critical for obtaining healthy and high-quality seeds. However, the treatment of post-harvest seeds is also prevalent for controlling seed-borne diseases (Hertwig et al., 2018). Seed treatment for preventing pathogenic diseases focuses on the inactivation of pathogens that are present on the surface and inside of infected seeds. Chemical-based microbicides have been frequently used in the decontamination of seeds, but alternative methods have also been attempted to complement the disadvantages of chemical control tools.

Application of atmospheric-pressure non-thermal plasma to control seed-borne diseases has been mostly performed by exposing the seeds to direct plasma, plasma-generated gas, or plasma-treated water. A significant number of studies have demonstrated that plasma and plasma-originated gas or water can effectively reduce the microbial load in seeds used either as food or for crop production (see **Tables 1** and **2**). To prevent seed-borne diseases, pathogenic microorganisms contaminating seeds should be inactivated. Many studies demonstrated the plasma-mediated removal of pathogenic microbes from the seed surface (see **Tables 1** and **2**). In these studies, the direct plasma treatment was most frequently performed on seeds. Plasma-generated gas (Los et al., 2018) and plasma discharged in water (Kang et al., 2015) have also been used to disinfect seeds. In addition, low-pressure plasma treatment showed the efficient removal of pathogenic bacteria from the vegetable seeds (Nishioka et al., 2016).

Despite its efficiency, seed disinfection by plasma demonstrates several issues that need to be solved. First, the standardization of the plasma dose for effective seed disinfection is needed. The plasma treatment unit has recently become a hot topic in plasma bioscience research community (Fridman et al., 2018). Treatment time is not enough to be a standardized unit because plasma sources and parameters vary among research groups. In addition, plasma generates various components, such as reactive species, ions, UV, and electrons, and the level of these components can be greatly affected by surrounding environmental condition. Therefore, the definition of a plasma dose in common terms should be established for fine-tuning plasma application. Second, there is still a great need for experimental evidences on the vitality of the germinated seedlings or plants from plasma-treated seeds. Several studies have reported an improvement in seed germination as well as microbial disinfection after plasma treatment (Mitra et al., 2014; Kordas et al., 2015; Khamsen et al., 2016; Stepanova et al., 2017). This might be a consequence of the removal of pathogenic microbes or activation of the seed germination process by plasma itself (Randeniya and de Groot, 2015). In many studies, plasma treatment demonstrated no adverse effects on seed vitality (such as germination and subsequent growth) at antimicrobial doses (Selcuk et al., 2008; Jo et al., 2014). These results indicate that the plasma treatment is a quite promising technique for seed disinfection.

The mechanisms of seed disinfection by plasma are not yet completely elucidated. Studies have demonstrated the harmful effects of plasma-generated reactive species, charged species, and UV photons on microbial cells (reviewed in Hertwig et al., 2018). Radicals and excited molecules generated from plasma can erode the surface of microbial cells through etching and, thus, lead to their inactivation (Moisan et al., 2001). Plasma-generated reactive species can cause oxidative damage to intracellular macromolecules, such as membrane lipids, proteins, and DNA, and a reduction in intracellular pH from diffusion into the microbial cells disrupting pH homeostasis (Laroussi and Leipold, 2004; Li et al., 2013). Microbial cell membranes can be electrostatically disrupted through the accumulation of charged species on the surface, and UV photons from plasma can induce DNA damage (Laroussi, 2002). Although the plasma-mediated inactivation of microbes on the seed surface is likely caused by the adverse effects of plasma on microbial cells, additional factors, such as the interaction between plasma and the seed surface, should be also considered. The physicochemical changes occurring on the seed surface can cause the damage on microbes (Mitra et al., 2014). More intense studies are needed to elucidate the mechanisms underlying microbial inactivation.

### Foliage and Root Diseases

Foliage and root diseases are some of the most serious plant diseases in agriculture because of the difficulties in controlling them. Leaf and root infection with bacterial and fungal pathogens might cause a serious reduction in the quality and yield of plants, particularly of leafy and root vegetables. Compared to seeds, leaves and roots are relatively more difficult to treat with plasma because they are possibly more liable to be damaged and changed by the plasma treatment (Zhang et al., 2014; Seol et al., 2017).

Spores of *F. oxysporum*, a pathogenic fungus infecting tomato root, can be inactivated after plasma treatment in saline (0.85% NaCl), as demonstrated by Panngom et al. (2014). However, plasma treatment on infected leaves or roots has rarely been reported. Zhang et al. (2014) reported that the plasma jet treatment of fungal infected spots on leaves resulted in the recovery of small-sized infected spots to the normal state. In this study, authors suggested a possible mode of plasma action by which the plasma-generated reactive species could penetrate leaf tissues through the stomata and inactivate fungal cells inside tissues (Zhang et al., 2014).

**Figure 1:**
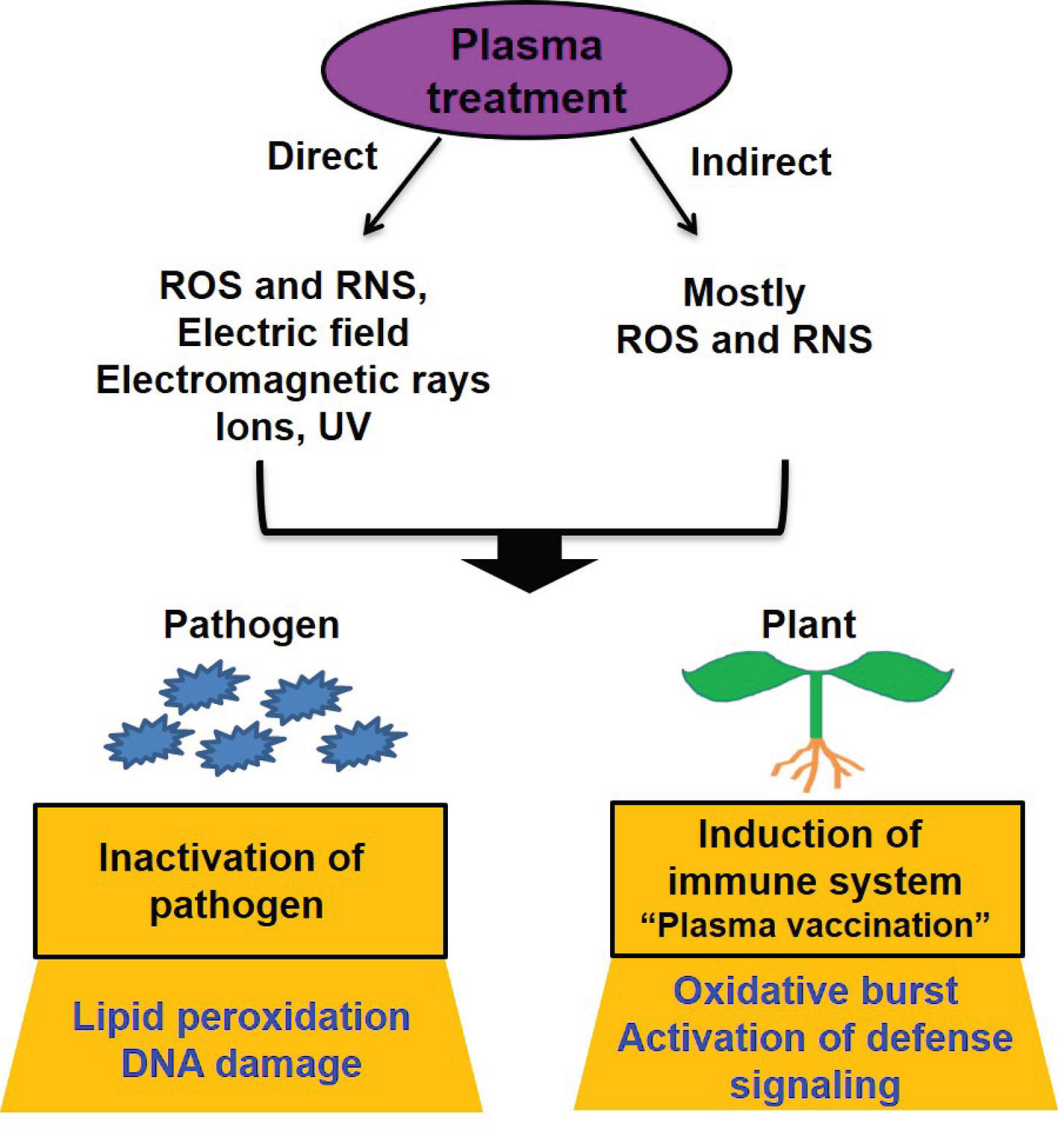
Models of plant disease control by plasma. Plasma can be applied directly or indirectly (plasma-treated water or media) to plants. Many plasma factors such as ROS, RNS, electric field, electromagnetic rays, active ions, and UV can be involved in disease control in direct plasma treatment whereas ROS and RNS from plasma are major players in indirect plasma treatment. Plasma (direct and indirect treatment) can inactivate pathogens associated with plants and seeds by causing membrane lipid peroxidation and DNA damage. In addition, it can be possible that plasma (direct and indirect treatment) induces plant immune responses by causing oxidative burst and continuously activating defense signaling, leading to the expression of defense genes.

Recently, a new direction has been added to plasma application for the control of foliage and root diseases—the induction of plant resistance by plasma, or so-called “plant vaccination” (**Figure 1**). The RONS are well-known signaling molecules regulating disease stresses in plants (Simontacchi et al., 2015; Dietz et al., 2016). Since plasma produces reactive species, the induction of disease tolerance and resistance can be possibly triggered by plasma treatment. Several studies have demonstrated experimental evidence supporting this notion. Jiang et al. (2014) observed that tomato seed germination and growth were increased after plasma treatment, and plants became more resistant to bacterial wilt disease in the leaves. After inoculation with a bacterial pathogen, the amount of H_2_O_2_ and the activities of resistant enzymes were increased more in plasma-treated plants than in untreated plants (Jiang et al., 2014). The application of PAW reduced the infection rate of *Xanthomonas vesicatoria* causing leaf spot disease in tomato plants to approximately 83% and increased the expression of disease resistance genes (Bertaccini et al., 2017). In addition, PAW effectively activated the grapevine defense response to phytoplasma (parasitic plant bacteria lacking cell walls) in a vineyard (Bertaccini et al., 2017), indicating the ability of PAW to induce disease resistance. Interestingly, Panngom et al. (2014) observed that the exposure of tomato plant leaves to non-thermal micro DBD plasma also induced an increase in the expression level of pathogenesis-related (PR) genes in the roots without significantly affecting plant growth. This indicates that plasma treatment of plant leaves can induce the development of defense barriers in other plant parts, such as the roots, possibly through defense hormone signaling.

### Post-Harvest Diseases

The microbial contamination of post-harvest crop products can occur during pre-harvest, harvest, transportation, storage, and distribution of products to the consumer. Post-harvest diseases lead to the spoilage of plant products used as food, resulting in great economic loss. Surprisingly, more than a third of fruits and vegetables become unavailable to the consumers because of the pathogenic infections (Oerke and Dehne, 2004). Alternative technologies are particularly needed for post-harvest disease control because traditional strategies, such as breeding for disease resistance and the use of chemical agents, have continuously generated problems.

Application of the non-thermal plasma technology for post-harvest disease control has been more often demonstrated in the inactivation of fungal pathogens contaminating vegetables and fruits (**Table 2**), probably because fungal infection and contamination are a major threat to post-harvest fruits and vegetables (Petriacq et al., 2018). Moreover, the contamination with fungal mycotoxins is a serious problem for food safety because they are very harmful to human and animal health. Fungal pathogens, such as *Alternaria, Aspergillus, Botrytis, Colletotrichum*, *Fusarium*, *Penicillium, Rhizopus*, and *Trichothecium*, predominantly contaminate post-harvest fruits and vegetables (Petriacq et al., 2018). As described in the earlier section, seed grains, such as rice, barley, wheat, and corn, can be efficiently disinfected by atmospheric-pressure non-thermal plasma without causing serious harmful effects on seeds. The contamination of hazelnuts, pistachio nuts, peanuts, and maize with *A. flavus* and *A. parasiticus* can be reduced through a low- or atmospheric-pressure cold plasma treatment (Basaran et al., 2008; Dasan et al., 2016a; Dasan et al., 2016b; Sohbatzadeh et al., 2016; Dasan et al., 2017). Fungal contamination on harvested fruits can be removed by treating them with plasma, as demonstrated in grape, banana, lemon, blueberries, date palm, and citrus fruits (Liu et al., 2011; Hayashi et al., 2014; Lacombe et al., 2015; Ouf et al., 2015; Yagyu et al., 2016; Won et al., 2017; Puligundla et al., 2018b). In most of these studies, fresh fruits were treated directly with plasma. However, Hayashi et al. (2014) treated lemons with active oxygen species generated by a combination of atmospheric-pressure plasma and UV light in ambient air. Liu et al. (2011) developed a special chamber equipped with a high-field plasma system under temperature and humidity control and demonstrated the longer preservation of grapes and bananas in this chamber without increasing bacterial and fungal infection on the fruit surface. In this system, there were no apparent discharges between electrodes and, therefore, the production of harmful factors, such as ozone, OH radicals, and UV, was suppressed. This study indicates that a high electric field generated from plasma could play a major role in fungal inactivation. Plasma can also be used to remove the fungal contamination on fresh vegetables, such as mung bean sprouts, and hence improve their shelf-life (Xiang et al., 2019). However, the fungal disinfection of fresh vegetables is not reported as frequently as that of fresh fruits.

The efficient sanitation of bacteria-contaminated fresh leafy vegetables by plasma is frequently reported, as illustrated in corn salad leaves, vegetable radicchio leaf, romaine lettuce, perilla, cabbage, spinach, and sprout vegetables (see **Table 1**). These studies also demonstrated that plant quality characteristics, such as pigmentation and leaf properties, are not significantly affected by the plasma treatment. Furthermore, the plasma treatment showed the efficient disinfection of harvested fruits, such as grape, banana, blueberry, tomato, strawberry, pepper, cantaloupe, and citrus, contaminated with *Bacillus*, *E. coli*, *Listeria*, *Salmonella*, and *Staphylococcus* (Liu et al., 2011; Niemira, 2012; Misra et al., 2014; Ziuzina et al., 2014; Hertwig et al., 2015; Jiang et al., 2017; Mošovská et al., 2018; Puligundla et al., 2018b). These studies showed that the efficiency of disinfection by plasma is different among species. Bermudez-Aguirre et al. (2013) compared the efficiency of bacteria removal by plasma among three fruits and vegetables, i.e. lettuce, carrots, and tomatoes, contaminated with *E. coli*, and found that tomato was disinfected more efficiently than lettuce and carrots. The researchers noted that the higher disinfection efficiency of tomato might be caused by its smooth surface structure (Bermudez-Aguirre et al., 2013). As demonstrated in many studies, the plasma treatment does not seem to damage the color and firmness of fruits and vegetables. Misra et al. (2014) demonstrated a 2-log_10_ reduction in bacterial load on strawberries inside a sealed package within 24 h after a 5-min treatment with plasma generated inside the package using ambient air (42% relative humidity), with no significant change in the color and firmness of strawberries. However, alterations at the cellular level, such as cell membrane irregularity, were observed (Bermudez-Aguirre et al., 2013). Dried fruits, such as dried figs, contaminated with bacteria were efficiently disinfected by plasma under low pH conditions because bacteria on the fruit surface were more sensitive to plasma treatment under the low pH conditions (Lee et al., 2015). Post-harvest almonds inoculated with *E. coli*, *Salmonella*, and *Shigella* have also been reportedly sanitized by the plasma treatment (Niemira, 2012; Fatemeh et al., 2018).

## Possible Mechanism(s) of Plasma Action in Disease Control

Non-thermal atmospheric-pressure plasma is well known for generating different types of RONS, along with UV, charged active species, electric field, and electromagnetic rays (Jha et al., 2017). Among these factors, RONS are most frequently stated as key players in microbial inactivation (Graves, 2012; Schmidt et al., 2017; Julák et al., 2018). Particularly, RONS are major components in plasma treated water or media considered as indirect plasma treatment (**Figure 1**). Plasma is generally known to generate reactive oxygen species (ROS), such as singlet oxygen, superoxide, ozone, and hydroxyl radical, and reactive nitrogen species, such as nitric oxide and nitric dioxide (Kim and Chung, 2016). Plasma might be used as an antimicrobial tool *via* the synergistic effect of various RONS and other physical factors. However, the composition of plasma-generated RONS can be varied depending on the used feeder gas (source), plasma device setting, and environmental conditions; thus, the efficiency of microbial inactivation can fluctuate (Girard et al., 2016). Each RONS can exert distinctive effects on microbial growth and plant growth and immunity (Turkan, 2018).

To understand the mechanism(s) of plasma-mediated disease control, the interaction of plasma-generated RONS with plant and microbial cells and their microenvironment should be established. The plasma-generated RONS can directly affect microbial cells or interact with the medium (buffer, growth media, or water) between the plant and microbial cells producing various RONS. The build-up of oxidative stress generated by RONS in the plant cells/pathogen microenvironment might eventually destroy microbial cells rather than plant cells because the threshold level of oxidative stress harmful to plant cells might be higher than that harmful to microbial cells (Dobrynin et al., 2009; Gay-Mimbrera et al., 2016). Although both plants and pathogens have antioxidant systems to alleviate oxidative stress, continuous or long-term exposure to plasma-generated RONS might enhance the level of intercellular RONS, which might overcome the antioxidant activity of microbial cells.

RONS play a differential role in plant–pathogen interactions. Their content upsurges in plants during the invasion of a pathogen. They either inactivate the pathogen growth or elicit the pathogen defense-related gene expression system (Shetty et al., 2008). Higher concentrations of ROS, such as superoxide, hydroxyl ion, and hydrogen peroxide, are toxic to pathogens and destroy them *via* oxidation of their macromolecules. At lower concentrations, RONS, such as NO and H_2_O_2_, act as signaling molecules to activate the defense signaling cascades and production of salicylic and jasmonic acids, inducing the systemic acquired resistance and hypersensitive reaction in infected plant parts (Torres et al., 2006).

### Direct Effect of RONS on Pathogens

Plasma-generated RONS are assumed to be the prime factors producing microbicidal effects (Dezest et al., 2017). These RONS, upon direct contact with a pathogen, affect its cell membrane. A short exposure to plasma leads to the loss of its culturability or pathogenicity, without affecting its metabolic activity (Dolezalova and Lukes, 2015). This VBNC state in pathogens might result from the initiation of cell membrane degradation by plasma-generated RONS. A longer plasma exposure or post-treatment incubation inactivates the pathogen up to 100% by disrupting the whole cell membrane (Joshi et al., 2011). Dezest et al. (2017) suggested the mechanism of plasma-induced bacterial inactivation by comparing the plasma treatments using three gases (He, He-N_2_, and He-O_2_) and different treatment methods. They observed that the He plasma treatment of PBS caused a greater inactivation of bacteria compared to that in the direct treatment. However, in the case of He-N_2_ plasma, both direct and indirect treatments of bacteria exerted bactericidal effects. In the case of He-O_2_ plasma, only direct treatment produced short-lived reactive species, such as ^1^O_2_, O_2_.^−^, and **^·^**OH, and exerts bactericidal activity. In liquids treated with He and He-N_2_ gas plasmas, H_2_O_2_ and NO are the major active components that produce bactericidal effects. Regarding the fungicidal mechanism of plasma, a study demonstrated that *F. oxysporum* spores might be inactivated by the peroxynitrite and hydroxyl radicles produced in saline treated with argon plasma (Panngom et al., 2014). Ochi et al. (2017) also showed that seed treatment with cold atmospheric plasma suppressed the progression of bakanae (*Fusarium fujikuroi*) and blight (*Burkholderia plantarii*) diseases in rice seedlings, and the ROS-mediated pathogen inactivation and immunity stimulation of rice seedlings might be responsible for disease suppression.

Plasma-generated RONS target proteins (intracellular and membrane proteins) rather than lipids. They modify a protein molecule by attaching to a carbonyl moiety (Weber et al., 2015). Several studies have reported that plasma-emitted RONS, charged particles, and electric waves could disrupt the membrane integrity of cells and penetrate them by causing surface abrasions in their membranes (Deng et al., 2010; Hensel et al., 2015; Wende et al., 2015). In a pathogen, the movement of ROS from environment through cell membrane causes oxidative stress, and the oxidation of cell regulatory macromolecules, such as proteins, nucleic acids, and lipids, ultimately leads to cell death (Winter et al., 2011; Lackmann and Bandow, 2014). Interestingly, Han et al. (2016) proposed the different modes of action of plasma-generated ROS on the inactivation of Gram-positive and Gram-negative bacteria. Gram-negative bacteria were inactivated mainly by cell leakage *via* cell envelope damage caused by plasma-generated ROS. In Gram-positive bacteria, significantly increased levels of intracellular ROS were observed with little envelope damage after plasma treatment, and high levels of ROS damage intracellular biomolecules, such as protein and nucleic acid, leading to bacterial inactivation. According to this study, the thickness of the peptidoglycan layer is not likely to influence greatly on the level of bacterial damage by plasma-generated ROS because both Gram-negative and Gram-positive bacteria are damaged at similar levels. Lipid peroxidation by plasma-generated ROS on the outer membrane lipopolysaccharides of Gram-negative bacteria resulted in breakage of the cell envelope, whereas the oxidation of peptidoglycan did not. This indicated that different structures of the bacterial cell wall may be critical for the mode—but not the level—of cell damage by plasma-generated ROS.

### Role of RNS and ROS in Plant Immunity

The plant–pathogen interaction is a complex and evolved process initiated at the time when pathogens try to invade the plant tissue. In this process, pathogens attempt to invade and proliferate, and plants try to identify them and activate their immune system. A major response of plants against pathogen invasion is to speed up the production of RONS in their cells (Fones and Preston, 2012). The accumulation of RONS inside plant cells (oxidative burst) excites their own antioxidant system and is toxic to the invading pathogens. These RONS not only destroy the pathogen, but also activate signaling cascades related to plant defense gene expression and hypersensitive response (Chris and Richard, 1997; Vanacker et al., 1998).

Plasma-generated RONS can enter the plant either through wounding, mechanical stress (in direct treatment), or small openings, such as the stomata. After entering the plant, they can be sensed by the plant cells, leading to the oscillation of intracellular RONS concentration. Unstable RONS, such as superoxide and singlet oxygen, might react with cellular components at close proximity, whereas stable RONS, such as H_2_O_2_ and NO, can move through the cell wall *via* diffusion. In the apoplast, the intracellular space between plasma membrane and cell wall, H_2_O_2_ and NO diffuse freely and interact with the cell membrane receptors. The apoplastic NO and H_2_O_2_ can move into the apoplast through aquaporins (Jang et al., 2012). Both NO and H_2_O_2_ act as signaling molecules and activate the expression of different defense-related genes, proteins, and hormones.

As a signaling molecule, hydrogen peroxide (H_2_O_2_) mainly targets Ca^2+^ homeostasis and alters ion channels, transcription factors, kinases, and phosphatases by oxidizing the methionine residues of proteins (Ghesquière et al., 2011; Petrov and Van Breusegem, 2012). The mitogen-activated protein kinase (MAPK) cascade, which is activated during plant–pathogen interactions, is dependent on the H_2_O_2_ signal (Zhang et al., 2007; Xing et al., 2008). The MAPK enzyme phosphorylates many transcription factors, such as WRKY and ANAC042. Transcription factors, such as NAC, ZAT, DREB, bZIP, and MYB, are also activated by H_2_O_2_ signaling (Petrov and Van Breusegem, 2012). These activated transcription factors can be involved in regulating the expression of defense-related genes. H_2_O_2_ also activates the benzoic acid 2-hydroxylase enzyme, which catalyzes the synthesis of salicylic acid from benzoic acid. Salicylic acid triggers the systemically acquired response and induces PR gene expression in cells (Leon et al., 1995).

Another signaling species, nitric oxide, triggers a hypersensitive response, an evolved innate immune response to protect plants from pathogens and herbivores, by regulating jasmonic acid signaling cascade proteins through the nitrosylation and de-nitrosylation of tyrosine residue. Nitric oxide also modifies the conformation of NPR1 (nonexpressor of PR gene 1) by the S-nitrosylation of Cys156 (Tada et al., 2008). Other than activating the plant immune response, nitric oxide also regulates the virulence and developmental stages of fungal pathogens in plants (Arasimowicz-Jelonek and Floryszak-Wieczorek, 2016).

## Plasma Technology Transfer to Agricultural Industry

For the past few decades, plasma technology has been frequently used in electronics, surface modification, and the energy saving industry (Weltmann et al., 2019). Moreover, promising research on plasma technology applications in biological science and environmental conservation has gained both public and industry attention. Food security, food packaging, and resources conservation are important parts of the agriculture industry. Plasma technology has been proven to have potential in agricultural applications, such as enhancing seed germination, seed decontamination, physiological plant growth, plant disease control, soil remediation, biological and chemical decontamination, water and gas cleaning, and food packaging and sterilization (Brandenburg et al., 2019). However, only a few of these studies were further processed for transferring the technology to the industrial scale. In Germany, plasma-treated water has been proven to have the ability to decontaminate lettuce on an industrial scale (Andrasch et al., 2017). In Japan, the University of Iwate collaborated with Energy Support Corporation, Japan, to perform a field investigation into delaying fruit ripening during transportation by the decomposition of ethylene gas using DBD plasma (Takahashi et al., 2018). The primary challenges in the transfer of this technology from the lab to industry are the 1) technology efficacies, 2) scale-up and design of the technology according to the industry, 3) toxicology and dose, 4) regulatory approval and validation of the technology, and 5) consumer acceptance (Cullen et al., 2018).

Non-thermal plasma technology has shown greater efficacies in the food industry because it enhances the shelf-life of food products; maintains the quality of food products; enhances the chemical safety of food products; has low energy requirements and low operational and maintenance costs; and is ecofriendly being considered as a green technology. However, scaling up plasma technology for the industrial process without compromising plasma uniformity remains a significant challenge in the food industry. The regulation and validation of the plasma process has met various challenges in different industrial areas because of its multivariate, multi-timescale, time variance, and non-linear operations between different products. New studies on the cytotoxic effects of plasma-activated solutions (Adachi et al., 2015; Chen et al., 2016) raised concerns about product toxicity and the safe treatment dose of plasma. Although no specific legal and regulatory guidance exists for plasma technology, toxicity in food products after plasma treatment needs to be addressed before transferring technology to the industrial level. Another challenge for the use of plasma technology in the agriculture industry is consumer acceptance. Studies have shown that consumers have poor awareness of the plasma food processing technology. Therefore, the acceptance of plasma processed food by consumers depends on only the taste and sensory properties (Cullen et al., 2018).

## Pros and Cons of Plasma Technology in Plant Disease Control

Many studies have demonstrated that non-thermal atmospheric-pressure plasma is a potential tool for inactivating bacterial and fungal cells pathogenic to plants. As an alternative disease control tool, plasma has exhibited several advantages and disadvantages compared to traditional control tools. Plasma technology has much less possibility to induce resistance from pathogens, which is a major problem of chemical-based controls. In addition, plasma can efficiently inactive chemical-resistant pathogens as well as non-resistant pathogens because it has no selectivity on types of pathogens. Since many species generated by plasma are unstable reactive species, its impact on the environment is short-lived. Thus, it is considered as an environmentally safer tool. Plasma is relatively safer than ionizing radiations, such as gamma-rays and X-rays, because of its lower energy level. However, plasma also has a limitation that should be improved; the efficiency of plant decontamination and disinfection varies depending on the properties of the plasma device, treatment conditions, plant species, and microenvironment of the plant–pathogen system. Numerous intensive studies are still needed to evaluate the potential of plasma in plant disease control. In addition, the mode of plasma action in plant disinfection and decontamination must be continuously explored.

## Future Perspective: Plasma Vaccination

As demonstrated in studies, atmospheric-pressure non-thermal plasma can be efficient for controlling plant diseases by inactivating pathogens or activating plant immune response (**Figure 1**). Inactivation of phytopathogenic microorganisms by plasma has been actively explored, and enormous information usefully applicable to industry is available. Compared to pathogen inactivation, the activation of plant immune response by plasma, the so-called “plasma vaccination”, is currently gaining attention as an emerging strategy in plant disease control (**Figure 1**). Vaccination is a phenomenon of activating the immunity of mammalian cells by mild exposure to biological and chemical agents against diseases. Plants also have an innate immune system, which can be activated by exposure to chemical and biological molecules (Gorlach et al., 1996; Nicaise, 2014). Plasma-generated RONS can enter the plant through wounding or small openings and further penetrate plant cells. This may elevate the level of RONS in plant cells, which is a quite similar situation in plant cells undergoing a hypersensitive response. This, in turn, might activate the systemic immune response in plants. A periodic treatment with plasma might reinforce disease resistance in plants.

Extensive experimental data demonstrating the possibility of plasma vaccination have not yet been accumulated. However, there have been occasional reports showing induction of plant disease tolerance by plasma at organismal and molecular level (Jiang et al., 2014; Panngom et al., 2014; Bertaccini et al., 2017). Recently, a study has also demonstrated that the priming of tomato seeds with plasma-treated water has elevated the level of intracellular RONS, defense hormones (salicylic acid and jasmonic acid), and transcripts of PR genes (Adhikari et al., 2019). In these studies, plasma and plasma-treated water generate no adverse effects on plant vitality. This indicates that plasma or plasma-treated water can possibly act as a trigger for inducing plant defense responses with no harm to plant vitality. Although research on plasma vaccination is in its initial stages, the potential of a new control strategy using plasma is quite promising. The pressure of plant diseases on agriculture has recently increased, particularly because of global warming. Thus, the induction of plant tolerance can be an important and environmentally safe strategy that should be intensively studied in the future.

## Author Contributions

BA, KP, and GP wrote the main manuscript and MV and SM helped in the literature search and summary. All authors reviewed the manuscript.

## Funding

This work was supported by the R&D Program of ‘Plasma Advanced Technology for Agriculture and Food (Plasma Farming)’ through the National Fusion Research Institute of Korea (NFRI), funded by government funds. The work was partially supported by the National Research Foundation of Korea (NRF) (2016R1D1A1B03934922) and by a grant from Kwangwoon University in 2019.

## Conflict of Interest

The authors declare that the research was conducted in the absence of any commercial or financial relationships that could be construed as a potential conflict of interest.
